# Digital health management models improve the metabolism, sleep, and gut microbiota in patients with metabolic disorders

**DOI:** 10.3389/fnut.2025.1631422

**Published:** 2025-09-25

**Authors:** Jie Zhang, Liang Ma, Qi Zhang, Hongmei Zhao, Luyan You, Jieru Zhou

**Affiliations:** Physical Examination and Health Management Center, Shanghai East Hospital, Tongji University School of Medicine, Shanghai, China

**Keywords:** lifestyle intervention, digital health management, metabolic disorders, visceral fat, chronic disease prevention

## Abstract

**Objective:**

This study aimed to explore the effects of lifestyle interventions based on a digital health management (DHM) model on metabolism, sleep, and gut microbiota in patients with metabolic disorders.

**Methods:**

This study enrolled 240 patients aged 18–65 years with at least one metabolic abnormality, who were randomized into the DHM group (*n* = 120) and control group (*n* = 120). The DHM group used a closed-loop digital management system consisting of the “Health Assistant” WeChat applet and the Huawei Band 7. This system enabled real-time data synchronization to deliver personalized dietary plans (calorie-targeted, adjusted for baseline metabolic parameters), dynamic exercise prescriptions (heart rate- and activity-adjusted with real-time feedback), and a sleep optimization module (white noise playback and breathing exercise prompts). The control group received conventional health education. The primary endpoint was the change in visceral fat area (VFA) over 12 months; secondary endpoints included the coefficient of variation (CV) of fasting blood glucose, resting energy expenditure (REE), brachial-ankle pulse wave velocity (baPWV) for arterial stiffness, gut microbiota abundance, and sleep quality scores.

**Results:**

After 12 months, the DHM group showed a significant reduction in VFA (from 122.9cm^2^ to 75.7cm^2^), with lower VFA than the control group at 3, 6, and 12 months (*p* < 0.05). In the DHM group, the CV of fasting blood glucose decreased to 8.4 ± 1.1% (*p* < 0.001), REE increased by 167 kcal/d (*p* < 0.001), baPWV decreased by 348.6 cm/s (*p* < 0.001), the abundance of butyrate-producing bacteria increased 3.1-fold (*p* < 0.001), and sleep quality scores improved to 93.1 ± 9.3 points (*p* < 0.001). All outcomes in the DHM group were significantly superior to those in the control group (all *p* < 0.05).

**Conclusion:**

The DHM model effectively improves body composition, glycemic stability, and cardiovascular risk in patients with metabolic abnormalities through multidimensional interventions, providing an evidence-based practical solution to chronic disease prevention.

## 1 Introduction

Metabolic disorders (MetDs) are a broad category of conditions including central obesity, hypertension, hyperglycemia, and dyslipidemia. In recent times, MetDs have become a major global public health challenge. According to the World Health Organization, approximately 25% of adults worldwide are affected by MetDs (1). The excessive accumulation of visceral fat is the core pathological basis of MetDs. It induces insulin resistance through lipotoxicity and releases pro-inflammatory factors that accelerate atherosclerosis (2, 3). Currently, lifestyle interventions, including diet control, exercise, and sleep management, are recommended as the first-line approach. Traditional health education models, however, face significant limitations: Guidance is often static, relying on paper materials or lectures, and fails to address individuals’ dynamic needs. Consequently, patient compliance remains low, typically below 40% (4). Although pharmacological interventions (e.g., statins and GLP-1 agonists) and surgical interventions can rapidly improve metabolic parameters, their limitations restrict widespread application. GLP-1 agonists may cause gastrointestinal adverse effects, including nausea and diarrhea, in up to 40% of users, while bariatric surgery carries a 2–5% risk of anastomotic leaks and costs more than USD$15,000 per procedure in many regions (5). Thus, there is an urgent need for an efficient, sustainable, and personalized model to prevent and control MetDs.

In recent years, digital health management (DHM) has emerged as a promising solution to these challenges. Mobile health applications and wearable devices enable continuous, real-time tracking of dietary intake, physical activity, and physiological parameters (6). For instance, continuous glucose monitoring technology provides dynamic feedback on postprandial blood glucose fluctuations, guiding precise nutritional adjustments. Meanwhile, smart wristbands analyze heart rate variability (HRV) to optimize exercise prescriptions (7). However, the existing research still has the following limitations. First, functionality is often singular, with most platforms lacking integrated mechanisms to coordinate diet, exercise, and sleep interventions. Second, algorithmic personalization is limited, reducing intervention precision. Long-term effectiveness data are scarce, hindering the provision of reliable evidence for clinical decision-making (8).

To address these shortcomings, this study innovatively constructs a “closed-loop DHM model,” defined as a system in which real-time patient data from wearables and apps are continuously analyzed, and interventions are dynamically adjusted based on generated insights. This closed-loop design integrates wearable devices and multi-omics analysis techniques, aiming to overcome the limitations of traditional interventions. At the technical level, this study could personalize the dynamic management of diet and caloric budgeting systems, optimize exercise prescriptions, and integrate data synchronization from multiple platforms. From a clinical perspective, this study systematically evaluates the multidimensional impacts of DHM on visceral fat, blood glucose fluctuations, arterial stiffness, and autonomic nervous function through a 12-month randomized controlled trial. Additionally, the developed health management tools can be directly applied in community and primary medical settings, supporting the implementation of the “Healthy China 2030” strategy.

## 2 Methods

### 2.1 Patient selection

The study was conducted at the Health Checkup Center of our hospital from January 2023 to January 2024. The inclusion criteria for the patients were as follows: (1) having at least one of these conditions: hypertension (untreated office blood pressure ≥140/90 mmHg, or diagnosed and receiving antihypertensive treatment), hyperglycemia (fasting blood glucose ≥6.1 mmol/L or HbA1c ≥ 5.7%, or diagnosed with diabetes), hyperlipidemia (LDL-C ≥ 3.4 mmol/L, or TG ≥ 1.7 mmol/L, or diagnosed with dyslipidemia); (2) aged 18–65 years; and (3) ability to independently use the WeChat applet and Huawei Band 7, with a commitment to daily usage of ≥30 min. Patients were excluded if they had: (1) severe cardiovascular or cerebrovascular events in the past half year, (2) chronic diabetic complications, (3) pregnancy or plan to become pregnant within 1 year, (4) breastfeeding, (5) alcohol dependence, (6) mental illnesses, (7) color blindness or visual impairment, and (8) hand deformities preventing the wearing of the wristband.

During the study, patients were withdrawn under the following conditions: (1) severe adverse reactions (e.g., hypoglycemic events exceeding three times/month), (2) persistent compliance of less than 50% for 4 weeks, and (3) an active request to withdraw from the study.

The primary endpoint of this study was the change in visceral fat area (VFA) at 12 months, with an expected decrease of 25 cm^2^ in the DHM group and 5 cm^2^ in the control group. Based on parameters (*α* = 0.05, *β* = 0.1, standard deviation = 15 cm^2^) and a dropout rate of 15%, a minimum of 108 participants were required in each group.

This study was approved by the Medical Science Committee and Ethics Committee of our hospital (No.: SE2023016-C-1). All research procedures strictly adhered to the Declaration of Helsinki. Written informed consent was provided by all participants prior to enrollment.

### 2.2 Intervention measures

Patients in the control group implemented regular health management, including a 60-min health education lecture every 3 months. The lectures were based on the Dietary Guidelines for Chinese Residents, focusing on topics such as weight management, dietary principles, and precautions for managing blood sugar, lipids, blood pressure, exercise plans, safety recommendations, strategies for continuous lifestyle improvement, and relapse prevention. Monthly telephone follow-ups were conducted to record their dietary and exercise conditions and reinforce adherence to guidance.

The DHM group utilized a WeChat applet named “Health Assistant” integrated with Huawei Band 7, featuring smart dietary management, exercise prescription adjustments, and sleep optimization. Patients accessed the applet to fill in baseline information. Based on the analysis of data from smart devices, patients should implement personalized management.

### 2.3 Daily caloric needs

Daily caloric needs were calculated based on the Guidelines for Medical Nutrition Therapy for Overweight/Obese Patients in China (2021) and the Adult Obesity Management Guidelines from the National Heart, Lung, and Blood Institute of the USA (9–11). Basal metabolic rate (BMR) was calculated using the Mifflin-St Jeor formula recommended by the American Dietetic Association, with male BMR = 10 × weight (kg) + 6.25 × height (cm) − 5 × age (y) + 5 and female BMR = 10 × weight (kg) + 6.25 × height (cm) − 5 × age (y) − 161 (12). The total daily energy expenditure (TDEE), calculated as BMR multiplied by an activity factor, was adjusted based on individual activity levels, as detailed in Table 1. The calorie deficit was determined based on individual BMI values. For those with a BMI beyond 24, TDEE was reduced by 500–750 kcal (to achieve a weight loss of 0.5–1.0 kg per week). For individuals with a BMI of 18.5–23.9, keep TDEE within ±10%.

**Table 1:** Comparison of baseline data between the two groups of patients.

Characteristics	DHM group (*n* = 120)	Control group (*n* = 120)
Sex, *n* (%)		
Male	56 (47)	67 (56)
Female	64 (53)	53 (44)
Age (years), mean (SD)	43.6 (11.0)	44.2 (9.7)
BMI (kg/m^2^), mean (SD)	24.2 (2.7)	24.1 (2.7)
Education, *n* (%)		
Elementary school and below	30 (25)	16 (13)
Junior school	14 (11)	52 (44)
Technical secondary school or high school	33 (28)	23 (19)
College degree and above	43 (36)	29 (24)
Baseline visceral fat area, mean (SD)	122.9 (15.9)	126.0 (16.4)
Baseline CV (%), mean (SD)	13.0 (2.1)	12.3 (2.2)
Baseline REE (kcal/d), mean (SD)	1514.8 (199.6)	1505.0 (207.6)
Baseline baPWV, mean (SD)	1441.2 (188.4)	1439.1 (181.3)
Baseline relative abundance of intestinal butyrate-producing bacteria, mean (SD)	1.02 (0.21)	0.98 (0.20)
Baseline sleep score	66.5 (12.8)	69.1 (11.5)
Baseline deep sleep duration, mean (SD)	63.6 (14.4)	64.1 (13.4)
Baseline resting heart rate (bpm), mean (SD)	72.9 (6.9)	73.2 (6.5)
Baseline HRV (ms), mean (SD)	38.7 (6.4)	38.6 (6.0)
Baseline GSES (min), mean (SD)	5.2 (1.1)	5.1 (1.0)

### 2.4 Target heart rate and sleeping time

The real-time HR was detected by Huawei Band 7, and the target HRs were determined by referring to Guidelines for Exercise Testing and Prescription (11th Edition) and Physical Activity Guidelines (13). The specific calculation formulas are as follows: (1) the maximum heart rate (HRmax) = 211–0.64 × age and (2) the target heart rate zone using the Karvonen formula: Target heart rate = [(HRmax - resting heart rate) × intensity%] + resting heart rate (14). For moderate-intensity exercise, the target heart rate was set at 40–59% of HRmax, and for high-intensity exercise, the target heart rate was set at 60–89% of HRmax. Referring to the Sleep Duration Recommendations of the American National Sleep Foundation, patients were recommended to have 7–9 h of sleep (15).

### 2.5 The daily implementation

#### 2.5.1 Morning (7:00–8:00)

Patients should check data from the wearable device, including the night’s sleep score, resting heart rate, and HRV, and accomplish the daily tasks prompted by applet, such as consuming more than 200 g of leafy vegetables today, performing a brisk walk of 30 min at 4 PM, or maintaining a target heart rate of 120 bpm while walking or exercising.

#### 2.5.2 Five minutes before each meal

Patients should use the applet to photograph meals, with AI recognizing and calculating the calories of the ingredients. For unrecognized ingredients, a dropdown menu can be used for selection. If the estimated calories exceed the target value by 15%, a warning will pop up, such as, “It is recommended to reduce staple foods by 50 g and increase broccoli by 200 g.”

#### 2.5.3 Exercise

The wearable device automatically monitored the heart rate. If the real-time HR remained below the target HR for 5 min, a vibration alert will remind them.

#### 2.5.4 Sleep intervention

The sleep module of the applet could play white noise before sleep, and the wearable device entered silent mode upon detecting sleep. If sleep efficiency was less than 80% for 3 consecutive days, the coach mode would recommend breathing exercises.

### 2.6 Weekly task feedback

Every Sunday at 8:00 PM, the applet generated a weekly report that included dietary analysis for the week, exercise summary, and sleep improvement situations. The report was presented to patients and sent to the healthcare team online. Missing data were defined as non-compliance, and the researchers collected compliance data from the back end. For patients with less than 70% compliance, manual telephone intervention will be triggered.

### 2.7 Quality control measures

Researchers must complete at least 20 h of training on DHM courses and engage in relevant training. All participant data were encrypted and stored securely; personnel accessing data received training on legal and regulatory compliance. Participants retained the right to delete or export their data.

### 2.8 Observation indicators and assessment standard

As the core pathological driver, visceral fat releases pro-inflammatory cytokines and free fatty acids, including insulin resistance and atherosclerosis. Therefore, the reduction of VFA is directly correlated with improved metabolic homeostasis. The VFA was measured using a Siemens SOMATOM Definition AS 64-slice spiral CT scanner. Patients should fast for 8 h, lie supine with both arms raised, and be scanned from the diaphragm to the pubic symphysis. Settings include voltage of 120KV, current of 200 mA, slice thickness of 5 mm, and reconstruction slice thickness of 1 mm. AI segmentation software will outline the VFA in the transverse images at the level of the patient’s navel and perform calculations. Meanwhile, the body fat percentage was assessed via bioelectrical impedance analysis (InBody 770).

The coefficient of variation (CV) of fasting blood glucose was recorded to evaluate short-term glycemic instability. It utilized the FreeStyle Libre 2 continuous glucose monitoring system for 14 days of continuous monitoring after disinfecting the skin on the upper arm and attaching the sensor.

The resting energy expenditure (REE), correlated with energy balance, was measured using the COSMED Quark CPET metabolic chamber (Italy). Patients should fast for 12 h, rest supine for 30 min, then wear a breathing mask and monitor oxygen consumption and carbon dioxide production continuously for 20 min. Using the Weir formula, the REE was calculated.

Arterial stiffness was assessed by brachial-ankle pulse wave velocity (baPWV) and blood pressure using the Omron BP-203RPEIII device. Patients should be in a supine position, with cuffs placed on the upper arm and ankle for the simultaneous measurement of blood pressure and electrocardiograph, which was automatically calculated as the distance between brachial and ankle sensors divided by pulse wave transit time.

The relative abundance of butyrate-producing bacteria was detected using the Illumina NovaSeq 6,000 sequencing platform (USA). After morning stool collection, a sterile stool collection tube was used to gather samples. DNA was extracted using the QIAamp Fast DNA Stool Mini Kit and analyzed with MetaPhlAn4 for calculating the relative abundance of butyrate-producing bacteria.

Sleep situation was evaluated using the sleep quality scoring method. The sleep quality score (0–100 points) consists of an objective score from devices along with a subjective score. The objective indicators referred to a specific scoring criterion (Supplementary Table S2). The subjective scale was adapted from the Pittsburgh Sleep Quality Index (PSQI) scale (Supplementary Table S3), excluding medication-use items and adding an assessment of the sleep environment, with Cronbach’s *α* = 0.83 and a correlation coefficient = 0.91 with the full PSQI. Total score = Objective score × 0.6 + Subjective score × 0.4. Scores ranging from 85 to 100 points indicate an excellent level, 70 to 84 points represent a good level, 55 to 69 points correspond to a moderate level, and 0 to 54 points signify a poor level. Validation (n = 50) showed an intraclass correlation coefficient of 0.89.

Resting HR and heart rate variability (HRV) were measured using the Huawei Band 7. HRV was the variation in time between heartbeats, which reflected autonomic nervous system activity. Self-efficacy scoring was assessed using the General Self-Efficacy Scale (GSES), consisting of 10 items, with a maximum score of 10, indicating higher self-efficacy with higher scores.

### 2.9 Statistical analysis

Data compilation was performed using Excel 2021, while statistical analysis was conducted with SPSS 22.0 in this study. An intention-to-treat (ITT) analysis was adopted for all primary and secondary outcomes. All randomized participants (*n* = 240) were included in the analysis, with missing data handled using the multiple imputation method (5 imputed datasets) to maintain the integrity of the randomized design (16). Continuous variables that followed a normal distribution were presented as mean ± standard deviation (mean ± SD), with 95% confidence intervals (CIs). Comparisons between the two groups were analyzed using independent sample t-tests, and effect sizes were calculated using Cohen’s d to quantify the magnitude of differences (with Cohen’s d < 0.2 indicating a trivial effect, 0.2–0.5 a small effect, 0.5–0.8 a moderate effect, and > 0.8 a large effect). Repeated measures ANOVA was used for within-group comparisons, categorical variables are expressed as rates, and the chi-square tests were applied for inter-group differences, setting a *p*-value of < 0.05 as statistically significant.

## 3 Nomenclature

**Figure 1:**
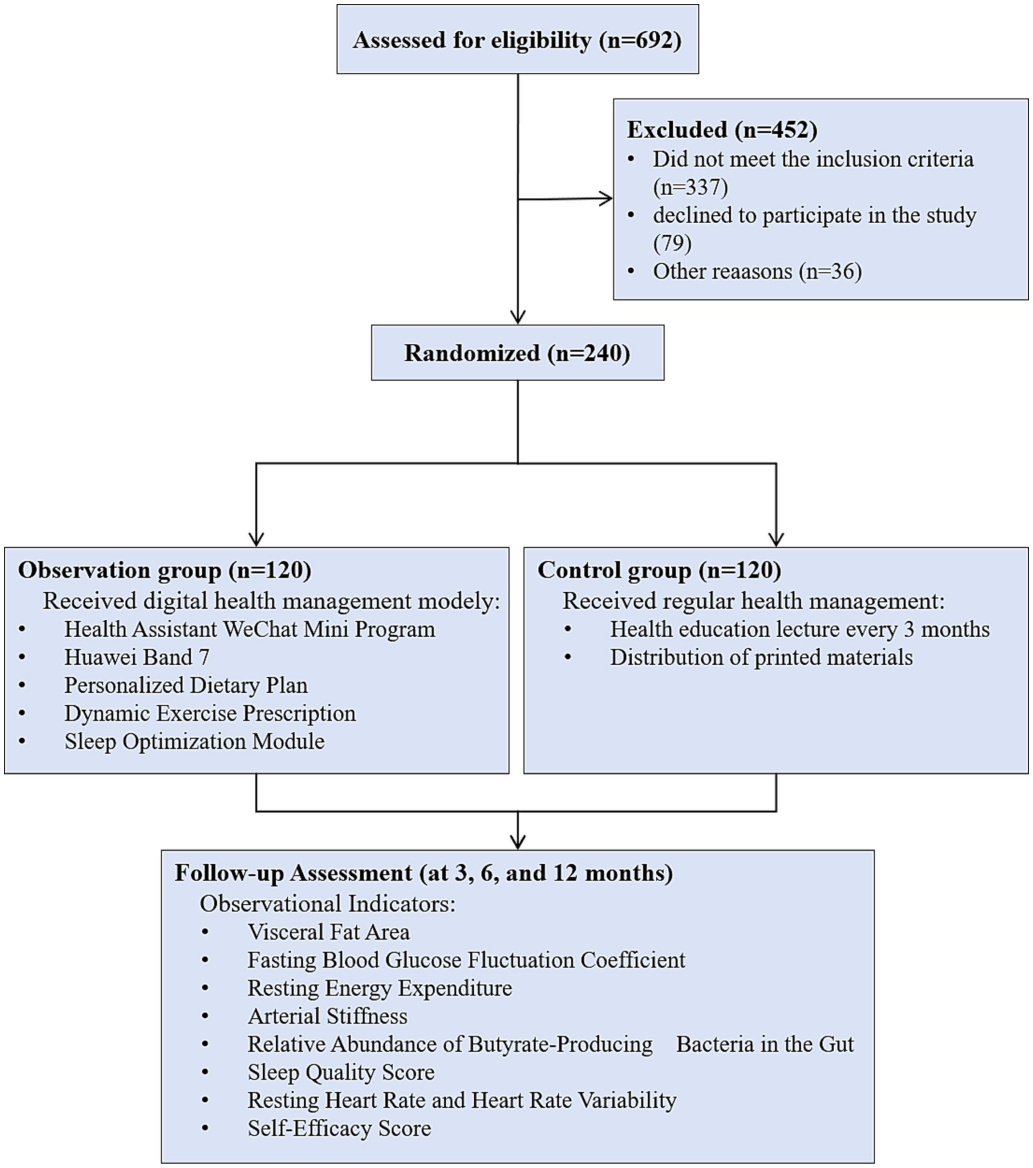
Study design flowchart.

First, the eligibility of 692 registered patients was assessed (Figure 1). We found that 337 patients did not meet the inclusion criteria, and 79 declined to participate in the study. Additionally, 39 patients were excluded from the study for other reasons. Ultimately, 240 patients were randomly assigned to the DHM group (*n* = 120) and the control group (*n* = 120). During the 3-month, 6-month, and 12-month intervention periods, no patients withdrew from the study in either group.

The baseline clinical data of patients in both groups, including gender, age, BMI index, and education level, were evaluated and compared for inter-group differences. The results indicated that there were no statistically significant differences in the data between the two groups (all *p* > 0.05) (Table 1).

### 3.1 Comparison of VFA and body fat percentage

**Figure 2:**
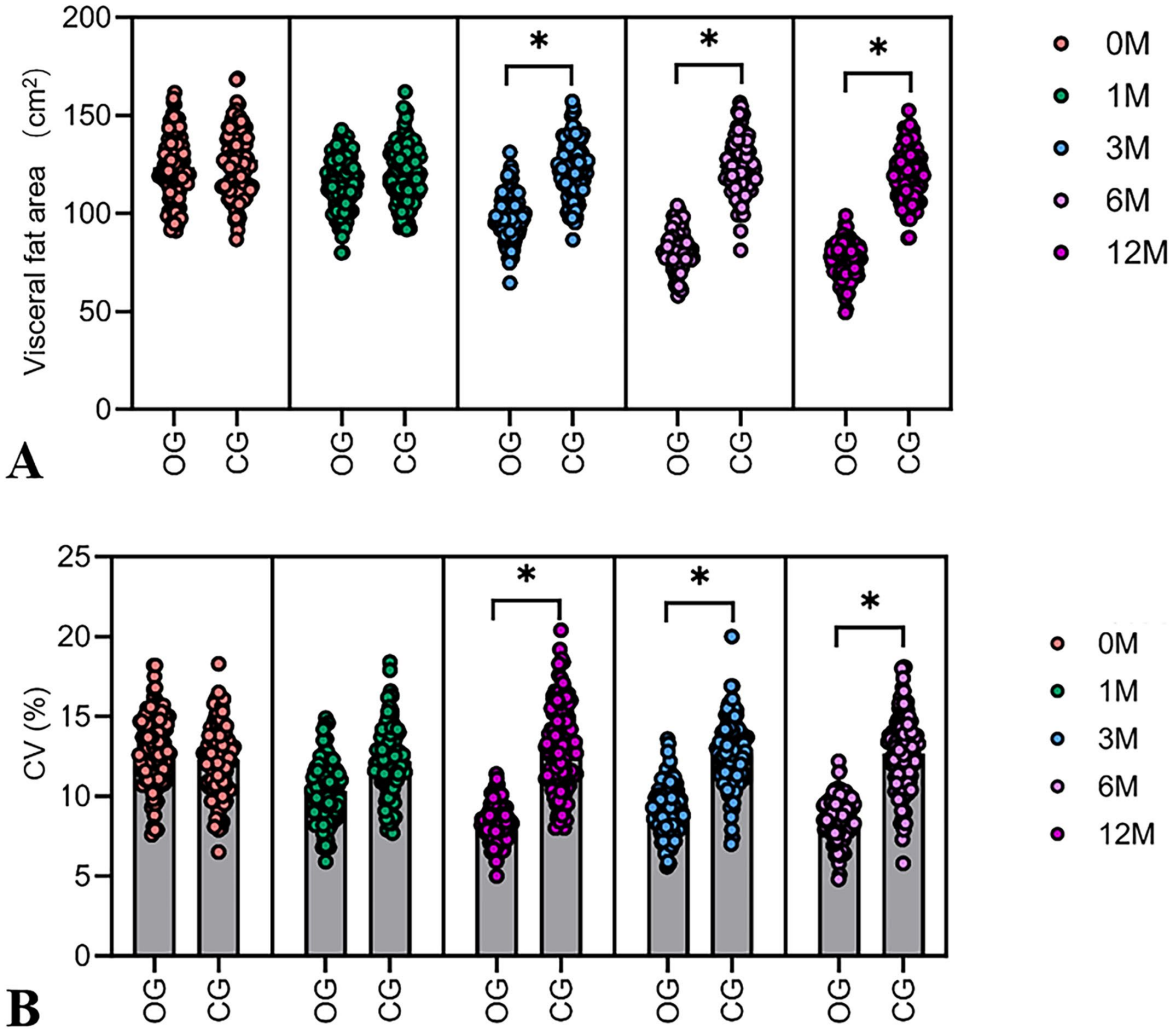
Dynamic changes in VFA and the CV of fasting blood glucose in two groups of patients. **(A)** The violin plot of VFA shows the difference in baseline visceral fat area between the DHM group and the control group. **(B)** The violin plot shows the difference in baseline CV between the DHM group and the control group. Compared with the control group, *p < 0.05.

Assessment showed that there were no statistically significant inter-group differences in VFA before the intervention. After 12 months of intervention, the VFA in the DHM group decreased by 47.2 cm^2^ (from 122.9 ± 15.9 cm^2^ to 75.7 ± 12.3 cm^2^), representing a 38.4% relative reduction (*p* < 0.001). In contrast, the control group showed a minimal decrease of 6.4 cm^2^ (from 126.0 ± 16.4 cm^2^ to 119.6 ± 15.8 cm^2^, 5.2% relative reduction). The DHM group had significantly lower VFA than the control group at 3 months (102.3 vs. 121.5 cm^2^, 15.8% relative difference), 6 months (89.1 vs. 120.2 cm^2^, 25.9% relative difference), and 12 months (75.7 vs. 119.6 cm^2^, 36.7% relative difference; all *p* < 0.05) (Figure 2A). The body fat percentage decreased from 41 ± 3.4% to 29.46 ± 4.6% in the DHM group, which was better than 33 ± 3.4% in the control group at 12 months (*p* = 0.001).

### 3.2 Dynamic changes in the CV of fasting blood glucose

The baseline CV showed no statistically significant inter-group differences. In the DHM group, the CV of fasting blood glucose decreased by 4.6 percentage points (from 13.0 ± 2.1% to 8.4 ± 1.1%), indicating a 35.4% relative reduction (*p* < 0.001). The control group showed a non-significant change (from 12.3 ± 2.2% to 11.8 ± 2.0, 4.1% relative reduction), resulting in a 31.3% relative difference between groups at 12 months (*p* < 0.05) (Figure 2B).

### 3.3 Changes in REE

**Figure 3:**
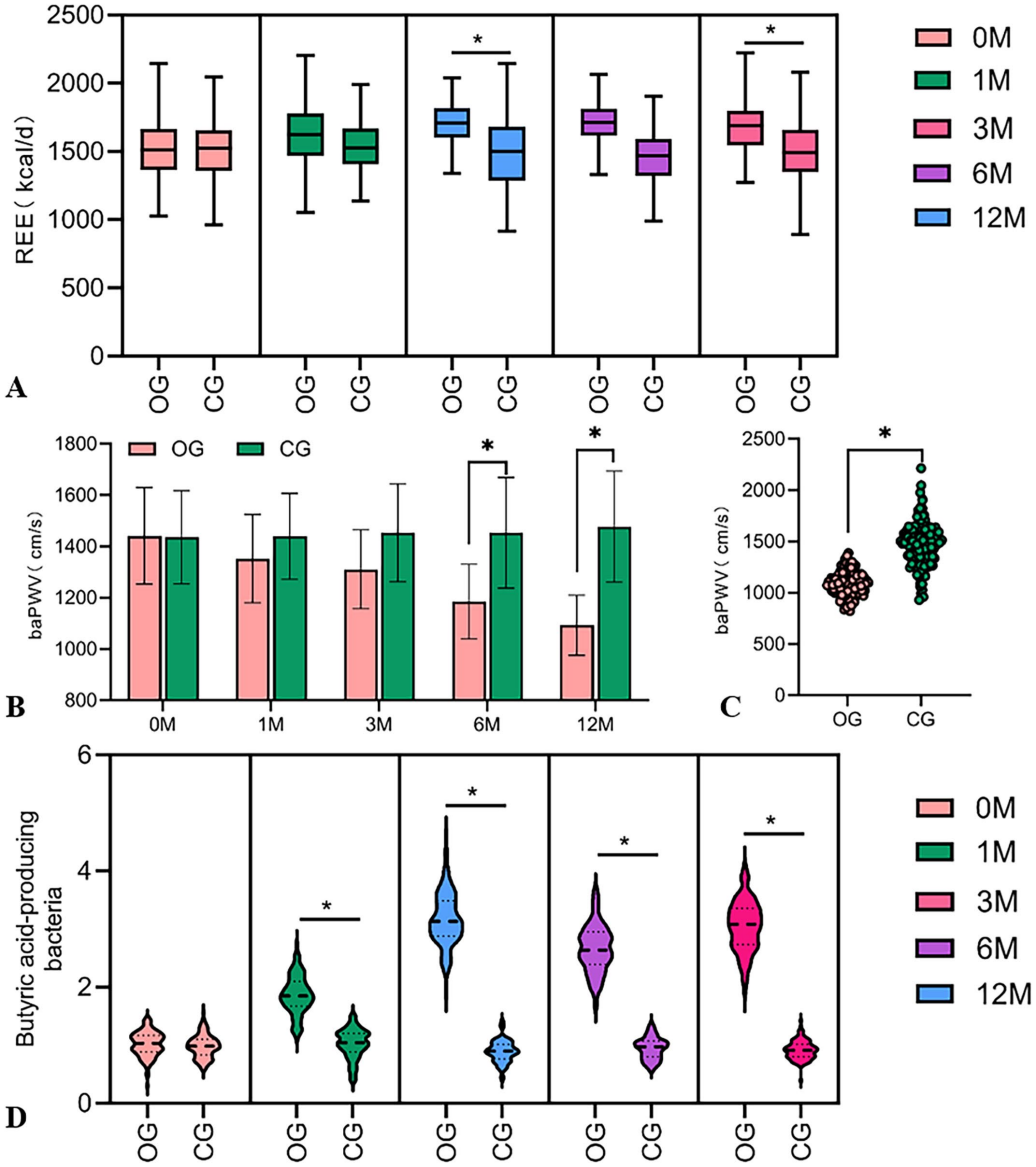
Comparison of REE changes, arterial stiffness, and relative abundance of butyric acid-producing bacteria in the intestines in two groups. **(A)** The violin plot shows the difference in baseline REE levels between the two groups. **(B)** The violin plot shows the difference in arterial stiffness after 6 months and 12 months. **(C)** After 12 months of intervention, arterial stiffness in the DHM group was lower than the baseline level. **(D)** The violin plot shows the difference in the baseline relative abundance of butyric acid-producing bacteria between the two groups. Compared with the control group, *p < 0.05.

The assessment revealed no significant differences in baseline REE between groups. REE in the DHM group increased by 167 kcal/day (from 1514.8 ± 199.6 to 1681.8 ± 210.3 kcal/d), with an 11.0% relative increase (*p* < 0.001), compared to a non-significant 15 kcal/day increase of REE in the control group (1.0% relative change) (Figure 3A).

### 3.4 The comparison of arterial stiffness

There was no statistically significant inter-group difference in baseline arterial stiffness. A baPWV in the DHM group decreased by 348.6 cm/s (from 1441.2 ± 188.4 to 1092.6 ± 165.2 cm/s), with a 24.2% relative reduction (*p* < 0.001), vs. a 42.3 cm/s decrease (2.9% relative reduction) in the control group (Figures 3B,C). Similarly, the blood pressure in the DHM group was decreased significantly compared to the control group (Table 2).

**Table 2:** Comparison of body indicators between the two groups of patients.

Characteristics	Group	*n*	Baseline	3 months	6 months	12 months
			Mean (SD)	Mean (SD)	Mean (SD)	Mean (SD)
HR^a^/bpm	DHM	120	72.9 (6.9)	69.6 (6.5)	67.7 (4.9)	66.4 (4.7)
	Control	120	73.2 (6.5)	71.5 (5.8)	71.4 (5.7)	72.5 (6.6)
	*P*-value	–	0.741	0.161	0.101	0.001
HRV^b^/ms	DHM	120	38.7 (6.4)	47.3 (8.3)	50.7 (6.9)	51.0 (8.4)
	Control	120	38.6 (6.0)	38.4 (6.5)	36.7 (5.5)	38.3 (8.9)
	*P*-value	-	0.507	0.079	0.023	0.846
Fasting blood glucose/mmol/L	DHM	120	12.95 (2.1)	10.35 (1.8)	9.23 (1.5)	8.65 (1.3)
	Control	120	12.31 (2.2)	12.21 (2.1)	12.73 (2.0)	12.68 (2.3)
	*P*-value	–	0.645	0.175	0.027	0.001
Systolic blood pressure/mmHg	DHM	120	182 (15.7)	171 (17.2)	157 (17.8)	146 (19.3)
	Control	120	183 (15.5)	175 (16.9)	169 (15.7)	164 (15.3)
	*P*-value	–	0.568	0.274	0.034	0.001
Diastolic blood pressure/mmHg	DHM	120	112 (12.5)	105 (11.9)	99 (11.5)	89 (10.5)
	Control	120	109 (12.6)	105 (12.5)	103 (13.3)	99 (11.9)
	*P*-value	–	0.759	0.109	0.038	0.002
Body fat percentage/%	DHM	120	41 (3.4)	36 (4.2)	32 (4.0)	29 (4.6)
	Control	120	40 (3.3)	37 (3.6)	35 (3.3)	33 (3.4)
	*P*-value	-	0.948	0.187	0.047	0.001

a HR, Heart rate.

b HRV, Heart rate variability.

### 3.5 The comparison of the relative abundance of gut butyrate-producing bacteria

There was no statistically significant difference in the baseline abundance of gut butyrate-producing bacteria between the two groups. The abundance of butyrate-producing bacteria in the DHM group increased by 3.1-fold (from 1.02 ± 0.21 to 3.16 ± 0.42), corresponding to a 210% relative increase (*p* < 0.001), with no significant change in the control group (1.05-fold, 5% relative increase) (Figure 3D).

### 3.6 The comparison of sleep conditions

There was no statistically significant inter-group difference in baseline sleep scores or deep sleep duration. At the 12-month follow-up after the intervention, sleep scores were higher in the DHM group compared to the control group (*p* < 0.05). At 3 months and 12 months after intervention, the deep sleep duration in the DHM group was significantly higher than that in the control group (*p* < 0.05) (Table 3).

**Table 3:** Sleep score situation and deep sleep duration between the two groups of patients.

Characteristics	Group	*n*	Baseline	3 months	6 months	12 months
			Mean (SD)	Mean (SD)	Mean (SD)	Mean (SD)
Sleep score	DHM	120	66.5 (12.8)	88.3 (12.5)	90.2 (10.9)	93.1 (9.3)
	Control	120	69.1 (11.5)	68.6 (9.5)	66.7 (9.1)	64.8 (12.2)
	*p*-value	–	0.232	0.764	0.273	<0.001
Deep sleep duration/minutes	DHM	120	63.6 (14.4)	88.3 (12.5)	90.2 (10.9)	93.1 (9.3)
	Control	120	64.1 (13.4)	63.8 (13.2)	64.7 (12.2)	62.3 (13.8)
	*p*-value	–	0.408	0.937	0.211	<0.001

### 3.7 Comparison of HR

No significant differences were observed between groups in terms of baseline resting heart rate or HRV. Twelve months after intervention, the resting heart rate in the DHM group was significantly lower than that in the control group (*p* < 0.05), and 6 months after intervention, HRV was elevated in the DHM group compared to the control group (*p* < 0.05) (Table 2).

### 3.8 Comparison of self-efficacy between both groups

There were no statistically significant inter-group differences in baseline GSES scores. At 1, 3, 6, and 12 months after intervention, the GSES scores in the DHM group were significantly higher than those in the control group (*p* < 0.05) (Table 4).

**Table 4:** Comparison of GSES scores between the two groups of patients (points).

Group	*n*	Baseline	1 months	3 months	6 months	12 months
		mean (SD)	mean (SD)	mean (SD)	mean (SD)	mean (SD)
DHM	120	5.2 (1.1)	5.5 (1.0)	7.9 (0.8)	8.6 (0.7)	8.7 (0.7)
Control	120	5.1 (1.0)	5.3 (1.2)	5.1 (1.2)	4.8 (1.3)	4.6 (1.4)
*p*-value	–	0.325	0.023	<0.001	<0.001	<0.001

### 3.9 Correlation analysis of CV improvement and REE increase

**Figure 4:**
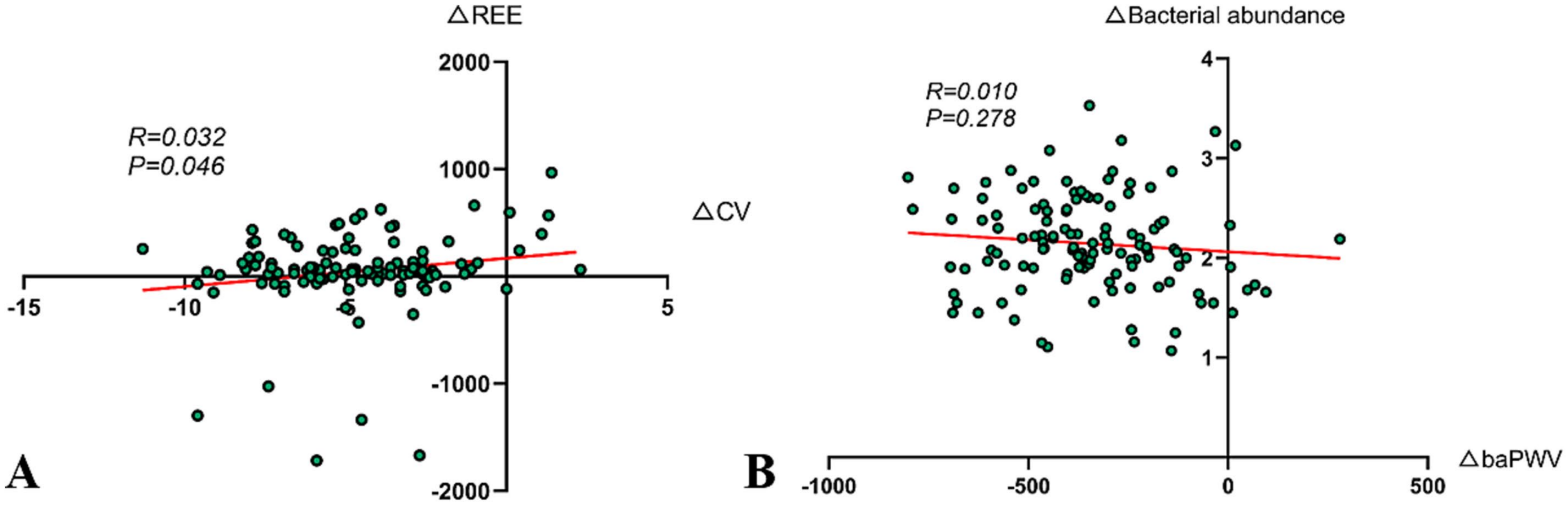
Correlation analysis. **(A)** Correlation analysis of improvement in CV and increase in REE (*r* = 0.032, *p* = 0.046). **(B)** Association between improvement in arterial stiffness and changes in the abundance of gut butyrate-producing bacteria (*r* = 0.010, *p* = 0.278).

The study separately calculated the differences in CV and REE from baseline levels in the DHM group at 12 months and performed a correlation analysis. The findings revealed a positive correlation between △CV and △REE (*r* = 0.032, *p* = 0.046) (Figure 4A).

### 3.10 Correlation between arterial stiffness improvement and gut butyrate-producing bacteria abundance change

The Pearson correlation analysis was used to explore the relationship between △baPWV and △gut butyrate-producing bacteria abundance in the DHM group at 12 months post-intervention. However, the results indicated that the correlation was not significant (*r* = 0.010, *p* = 0.278) (Figure 4B). In our experience, several factors might contribute to this result, including insufficient sample size, biological variability, short intervention period, dietary fiber intake, and baseline vascular calcification status.

## 4 Discussion

### 4.1 Research summary and key findings

This study systematically assessed the intervention effects of a DHM model on patients with metabolic abnormalities (including those with overweight/obesity and normal weight individuals with metabolic disorders) through a 12-month randomized controlled trial. Our results demonstrated that the DHM model yielded substantial improvements in key metabolic markers. VFA reduction exceeded 38%, CV improved by over a third, and these changes were sustained across 12 months (Figures 2A,B). These data suggest that the digital management model, by integrating dietary, exercise, and sleep interventions, can multidimensionally improve the pathophysiological state of patients with metabolic abnormalities, with effects significantly exceeding those of traditional health education.

### 4.2 Precise control of visceral fat reduction and metabolic benefits

In this study, the reduction in VFA in the DHM group was significantly greater than that in the control group, with a noticeable difference in fat reduction effects beginning from the 3-month intervention mark. Compared to existing studies, this study showed a significantly higher reduction in visceral fat (17). For example, Waters et al., in a study involving 160 obese older adult individuals undergoing aerobic and resistance exercise interventions, found that enhanced dietary and exercise guidance could improve participants’ physical functions, but the visceral fat reduction in that study was only 18% (18). Additionally, the Cunha team, through a randomized controlled and prospective study, found that a low-calorie diet could reduce liver fat scores, but the reduction was only 25% (19).

These two points represent the innovative aspects of this study. A literature review revealed that changes in the gut microbiota may also be related to improvements in VFA in the participants, as the butyrate-producing bacteria abundance in the DHM group increased approximately 3.1 times post-intervention (20). Butyrate-producing bacteria may activate the AMP-activated protein kinase (AMPK) pathway through short-chain fatty acids, inhibiting fat synthesis and promoting mitochondrial *β*-oxidation, as noted in research by the Pham team, which indicated that gut butyrate-producing bacteria help reduce abdominal fat expression by regulating fat deposition and oxidative stress via the Ffar2 pathway (21). Although the reduction in visceral fat is significant, other influencing factors cannot yet be completely ruled out, such as the role of gut microbiota changes in fat metabolism (22). This study shows that the increase in the abundance of butyrate-producing bacteria occurs simultaneously with the reduction of visceral fat, but the causal relationship between the two needs to be further clarified.

### 4.3 Dynamic control of blood glucose fluctuations

In this study, the CV for fasting blood glucose in the DHM group decreased from 13.0 to 8.4% (Figure 2B). Significant differences compared to the control group were evident starting 3-month post-intervention, with the DHM group showing a much greater improvement. The results of this study also surpass those of other similar studies (23). This significant difference may be attributed to the following technological integrations: (1) continuous glucose monitoring combined with image recognition for closed-loop management (24). Traditional studies often relied on self-reported dietary records, while this study combined Free Style Libre 2 continuous glucose monitoring with image recognition technology to achieve “meal component identification → blood glucose fluctuation prediction → real-time corrective suggestions.” For instance, when patients consumed high glycemic index foods, the system would push “walk for 15 min post-meal” suggestions based on continuous glucose monitoring trends to reduce post-meal peak blood glucose levels and (2) time-sequenced interventions between exercise and blood glucose (25). Early post-meal exercise can accelerate glucose uptake through skeletal muscle GLUT4 translocation, a mechanism termed the “exercise-glucose window” in other research; however, this study was the first to translate it into actionable behavioral interventions using digital tools (26).

Moreover, the improvement in CV was significantly positively correlated with the increase in REE (*r* = 0.032, *p* = 0.046), indicating that metabolic reprogramming may indirectly stabilize blood glucose by enhancing insulin sensitivity—providing new clinical evidence for the theory of metabolic flexibility (Figure 4A). However, the correlation coefficient suggests that this association, while statistically significant, is weak and requires validation through larger sample studies. Future research could integrate muscle metabolomics with continuous glucose monitoring data to further explore the regulatory mechanisms of digital interventions on skeletal muscle glucose metabolism and optimize the precise timing of post-meal exercise interventions to enhance blood sugar management efficiency.

### 4.4 Improvement in arterial stiffness and autonomic nerve regulation

The reduction in baPWV in the DHM group was 348.6 cm/s, which was markedly greater than that observed in the control group (Figures 3B,C). This advantage may stem from the synergistic effects of improved HRV and sleep regulation (27). HRV improvement is correlated with vascular relaxation function, as improved HRV in the DHM group suggests enhanced parasympathetic activity. Previous studies have confirmed that the parasympathetic nervous system releases acetylcholine, activating endothelial nitric oxide synthase and promoting vascular relaxation (28). Sleep regulation, through reduced sympathetic activity and diminished blood pressure fluctuations during deep sleep, may help reduce vascular shear stress damage (29). Furthermore, the authors propose that the reduction in baPWV may be related to the abundance of gut butyrate-producing bacteria. Previous studies have shown that butyrate can inhibit histone deacetylase, thereby upregulating Klotho protein expression, which is linked to anti-vascular calcification effects (30). However, the study’s results indicated weak correlation, possibly due to sample size or homogeneous sample sources, and future research will focus on this direction.

### 4.5 Interaction of the gut microbiota–sleep–metabolism axis

The abundance of butyrate-producing bacteria in the DHM group increased by 3.1 times, with significant differences evident from the first month of intervention. At the same time, sleep quality scores improved from 66.5 ± 12.8 to 93.1 ± 9.3 points. This significant change may involve sleep efficiency and microbial homeostasis (31). Deep sleep promotes the release of growth hormones, stimulates intestinal stem cell proliferation, and improves the colonization environment for gut microbiota (32). Additionally, the white noise used in this study may shorten sleep latency, increase the proportion of slow-wave sleep, and inhibit the excessive activation of the hypothalamic–pituitary–adrenal axis, maintaining the circadian rhythm of gut microbiota (33). Compared to existing research, the increase in butyrate-producing bacteria in the DHM group is comparable to the reported effects of dietary fiber interventions, but this study innovatively reveals the modulating effect of sleep quality on gut microbiota regulation. This finding enriches the research on the “gut-brain axis” and suggests that optimizing sleep may be an important entry point for regulating gut microbiota (34). Although the improvement in sleep and changes in gut microbiota were observed simultaneously, it remains difficult to establish a causal relationship between the two groups. Existing animal studies show that environmental noise interference can disrupt the circadian rhythm of gut microbiota, but evidence from human studies is still insufficient (35). Moreover, this study only assessed the abundance of butyrate-producing bacteria and did not comprehensively evaluate changes in overall microbial structure (36). Future research could combine polysomnography and metagenomic sequencing of gut microbiota to explore the bidirectional regulatory relationship between sleep structure and microbial composition in more detail and assess the regulatory effects of digital intervention programs on gut microbiota and metabolic parameters for different types of sleep disorders.

In summary, future research should focus on multi-center validation to confirm DHM model generalizability, long-term follow-up to assess the sustainability of metabolic improvements, and clarifying causal links between gut microbiota, sleep, and visceral fat. Integrating advanced omics could unravel the underlying mechanisms, while refining AI-driven tools for diverse diets may enhance efficacy, strengthening DHM’s role in chronic disease prevention.

## 5 Conclusion

This study confirms that the DHM model significantly outperforms traditional health education in improving metabolic disorders, with a 38.4% reduction in visceral fat area, a 34.6% decrease in fasting blood glucose variability, and a 3.1-fold increase in butyrate-producing gut bacteria over 12 months. These improvements translate into a reduced cardiovascular risk and a lower likelihood of diabetic complications, aligning with “Healthy China 2030” goals. While limited by a single center and short follow-up, the DHM model offers a scalable, evidence-based solution for chronic disease prevention. Future studies should explore the long-term sustainability of DHM effects and validate findings in multi-center and real-world settings to further support its widespread implementation in clinical and public health practice.

## Data Availability

The original contributions presented in the study are included in the article/Supplementary material, further inquiries can be directed to the corresponding author.
