# Supplementary material for: Digital health management models improve the metabolism, sleep, and gut microbiota in patients with metabolic disorders
Source: Front Nutr. 2025 Sep 25;12:1631422. doi: 10.3389/fnut.2025.1631422 (PMC12509696; doi:10.3389/fnut.2025.1631422)
Supplement: Supplementary file 1 [file Table_1.docx]

Supplementary Material

# Supplementary Tables

**Table S1. physical activity levels of different population groups.**

| Activity Level | Activity Factor | Example |
| --- | --- | --- |
| Sedentary (very little exercise) | 1.2 | Office workers with irregular exercise habits |
| Lightly active | 1.375 | Engaging in low-intensity exercise (like walking) 1-3 days a week |
| Moderately active | 1.55 | Engaging in moderate-intensity exercise 3-5 days a week |
| Heavily active | 1.725 | Manual laborers or athletes |

**Table S2. Objective indicators of sleep quality score.**

| Dimensions | Details | Power |
| --- | --- | --- |
| Total sleep duration | ≥7h (20 points），6-7h (15 points），5-6h(10 points），<5h (5 points） | 20% |
| Deep sleep ratio | ≥20% (20 points），15-20% (15 points），10-15% (10 points），<10% (5 points） | 20% |
| Sleep latency | ≤20min (10 points），20-30min (7 points），30-45min (4 points）, >45min (0 points） | 10% |
| Number of awakenings during the night | 0 (10 points），1 (7 points)，2 (4 points)，≥3 (0 points） | 10% |

**Table S3. Subjective indicators of sleep quality score.**

| Dimensions | Details | Power |
| --- | --- | --- |
| Self-Evaluation | 0-3 points (0=excellent, 3=poor) → converted to 0-10 points (reverse scoring) | 15% |
| Daytime Dysfunction | 0-3 points (0=None, 3=severe) → converted to 0-10 points (reverse scoring) | 15% |
| Sleep Environment Satisfaction | 0-3 points (0=very satisfied, 3=very dissatisfied) → converted to 0-10 points (reverse scoring) | 10% |
